# Metal-Insulator-Metal-Based Plasmonic Metamaterial Absorbers at Visible and Infrared Wavelengths: A Review

**DOI:** 10.3390/ma11030458

**Published:** 2018-03-20

**Authors:** Shinpei Ogawa, Masafumi Kimata

**Affiliations:** 1 Advanced Technology R&D Center, Mitsubishi Electric Corporation, 8-1-1 Tsukaguchi-Honmachi, Amagasaki, Hyogo 661-8661, Japan; 2 College of Science and Engineering, Ritsumeikan University, 1-1-1 Noji-higashi, Kusatsu, Shiga 525-8577, Japan; kimata@se.ritsumei.ac.jp

**Keywords:** plasmonics, metamaterials, metal-insulator-metal, absorbers

## Abstract

Electromagnetic wave absorbers have been investigated for many years with the aim of achieving high absorbance and tunability of both the absorption wavelength and the operation mode by geometrical control, small and thin absorber volume, and simple fabrication. There is particular interest in metal-insulator-metal-based plasmonic metamaterial absorbers (MIM-PMAs) due to their complete fulfillment of these demands. MIM-PMAs consist of top periodic micropatches, a middle dielectric layer, and a bottom reflector layer to generate strong localized surface plasmon resonance at absorption wavelengths. In particular, in the visible and infrared (IR) wavelength regions, a wide range of applications is expected, such as solar cells, refractive index sensors, optical camouflage, cloaking, optical switches, color pixels, thermal IR sensors, IR microscopy and gas sensing. The promising properties of MIM-PMAs are attributed to the simple plasmonic resonance localized at the top micropatch resonators formed by the MIMs. Here, various types of MIM-PMAs are reviewed in terms of their historical background, basic physics, operation mode design, and future challenges to clarify their underlying basic design principles and introduce various applications. The principles presented in this review paper can be applied to other wavelength regions such as the ultraviolet, terahertz, and microwave regions.

## 1. Introduction

Electromagnetic (EM) wave absorbers are drawing significant interest from aspects of both fundamental science and industry applications. Typical EM wave absorbers are essentially based on the intrinsic loss of the material and thus require a long optical path, which results in large volume and poor design flexibility. EM wave absorbers with absorption properties that can be efficiently controlled by their structures have thus been studied for many years. Such EM wave absorbers were first studied in the microwave range and are roughly classified into two groups, according to Reference [1], as broadband absorbers and resonant absorbers. The broadband absorbers are further categorized into two groups: geometric transition absorbers and low-density absorbers [1]. Geometric transition absorbers consist of two-dimensional (2D) periodic pyramids that cause a gradual change in the dielectric constant from the free space to the absorbers [2,3]. Low-density absorbers utilize porous materials [4,5] and the multi-reflections that occur in these pores has led to significant absorption, which was realized using thin absorbers.

**Figure 1:**
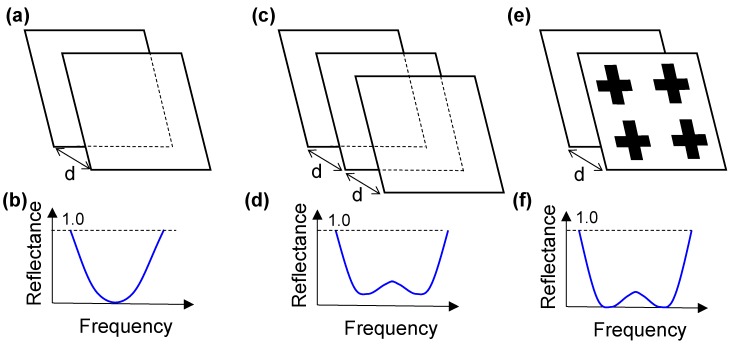
Schematic illustrations and reflectance of resonant absorbers: (**a**,**b**) the Salisbury screen; (**c**,**d**) the Jaumann absorber; and (**e**,**f**) the circuit analog CA absorber. “d” represents the quarter-wavelength gap [6].

The resonance absorbers are classified into three types, according to Reference [6]. Figure 1a–f shows schematic illustrations and the reflectance of the Salisbury screen, Jaumann absorber, and circuit analog (CA) absorber. All of these resonance absorbers use a quarter-wavelength gap from the top material to the bottom substrate. The Salisbury screen uses a non-periodic resistive sheet in front of a ground plate [7]. The Jaumann absorber uses two or more resistive sheets in front of each other and is a basic resonance absorber [8]. These two absorbers use purely resistive sheets. The CA absorber uses a periodic surface made of a lossy material with three layers: the top periodic metal patterns, a middle dielectric layer, and a continuous metallic bottom layer [6]. The concept of CA absorbers is the basis of recent metamaterial absorbers for a wide range of wavelength regions, from visible to microwave wavelengths.

Recent advances in plasmonics [9] and metamaterials [10] research together with the progress in nanotechnological fabrication techniques has led to novel EM absorbers at visible and infrared (IR) wavelengths. These absorbers uses localized surface plasmon polaritons (LSPPs) [11] with a metamaterial concept to achieve much smaller absorber volumes, sufficient performance, and design flexibility based on geometry rather than the materials used. SPPs are the collective oscillation of electrons between metals and dielectrics that can go beyond the diffraction limit [12]. LSPPs are key to realizing small and thin absorbers for the visible and IR wavelength regions. Therefore, much significant research has been performed on EM wave absorbers using SPPs or LSPPs at visible and IR wavelengths. 

There are roughly two categories of absorbers that employ plasmonics and metamaterials: conventional periodic structures such as plasmonic crystals [13,14,15] and gratings [16,17,18,19], and metamaterial-based structures, where periodicity has less impact on the optical properties [20]. In particular, metal-insulator-metal-based plasmonic metamaterial absorbers (MIM-PMAs) are the most promising and widely studied for a wide range of wavelengths due to their high performance, such as high absorbance, incident angle, and polarization insensitivity, as well as their design flexibility and simple fabrication. Although their fundamental principles are basically the same, a wide range of applications is expected, such as solar cells [21], refractive index sensors [22], optical camouflage [23], cloaking [24], optical switches [25], color pixels [26,27], thermal IR sensors [28,29,30,31], mechanical thermal sensors [32], surface-enhanced spectroscopy [33,34], and gas sensing [35]. Therefore, this review paper aims to clarify the fundamental principle, characteristics, possibilities, and challenges of MIM-PMAs at visible and IR wavelengths to contribute to future research and the expansion of their applications.

Please note that MIM-based thermal emitters are considered as MIM-PMAs at IR wavelengths [36] because absorbance is equal to emissivity, as given by Kirchhoff’s law. To the best of our knowledge, MIM-PMAs were first demonstrated as thermal IR emitters by Puscasu and Schaich [37].

## 2. Structures and Materials

In this section, the fundamental structures and materials of MIM-PMAs are explained with a simple introduction of the basic optical properties. The detailed optical characteristics are discussed in a later section.

**Figure 2:**
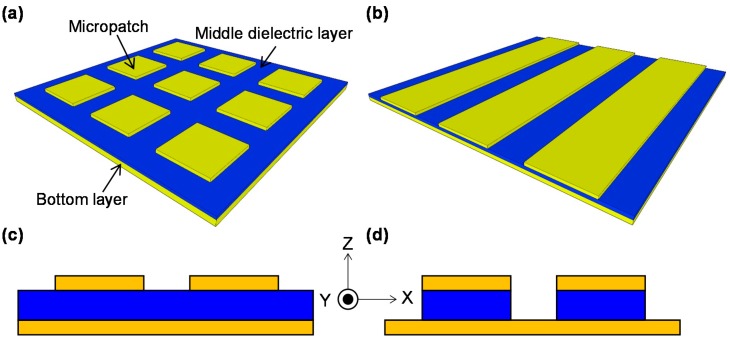
Schematic illustrations of metal-insulator-metal-based plasmonic metamaterial absorbers (MIM-PMAs); oblique view of (**a**) two-dimensional (2D) and (**b**) one-dimensional (1D) periodic micropatches. Cross-sectional views of (**c**) continuous and (**d**) isolated middle dielectric layers.

MIM-PMAs consist of three layers: a bottom metal layer, a middle dielectric layer, and a top periodic metal micropatches. Figure 2a,b shows schematic illustrations of conventional MIM-PMAs with two-dimensional (2D) and one-dimensional (1D) periodic micropatches, respectively. The absorption wavelength is fundamentally defined by the micropatch size. The 2D configuration is symmetric for two orthogonal directions, the *x* and *y* directions; therefore, the optical properties are polarization insensitive. On the other hand, the 1D configuration is asymmetric in the *x* and *y* directions, so the optical properties are polarization sensitive. Figure 2c,d shows cross-sectional views of the conventional MIM-PMAs with flat and isolated dielectric layers, respectively. The middle dielectric layers underneath the micropatches are required, so that both structures function as MIM-PMAs.

The thickness of the metal in the bottom layer and the top micropatches is required to be more than twice the depth of the operating wavelength region, e.g., 100 nm thickness is sufficient for the IR wavelength region [38]. The thickness of the middle dielectric layer can be less than the operating wavelength/50 due to the strong confinement of the waveguide mode of SPPs [39].

The possible lattice structures for 2D periodic micropatches are square, triangular, and honeycomb. However, the lattice structures have less impact on absorption properties because each micropatch acts as a single isolated resonator [40]. 

The shape of micropatches are roughly classified into symmetric in the two orthogonal directions, such as squares [28,41], circles [42], and crosses [38,43], and nano-particles [44,45,46], or asymmetric, such as ellipses [47], rectangles [48], wedges [49], bow-ties [50], split-ring resonators [51], and asymmetric crosses [52,53]. Nanocubes have also been used as micropatches [54,55]. The shape of the corners and the sidewall angles have an important role in defining single and multiband resonances [56]. The end shape of the micropatch produces the difference of optical modes formed in the middle dielectric layer because MIM structures can be considered as waveguides and the shape of the waveguide end defines the waveguide mode [57]. The first three micropatch shapes are symmetric in the *x* and *y* directions, and absorption occurs at a single wavelength. On the other hand, the latter four micropatch shapes are asymmetric in the *x* and *y* directions and produce two absorption wavelengths. As discussed in Section 4, the symmetry is an important parameter for polarization dependence.

It is also important to consider the temperature tolerance and compatibility of the complementary metal oxide semiconductor (CMOS) process for the choice of materials used for MIM-PMAs [43,58]. Table 1 and Table 2 show the properties of the metals and dielectrics used in MIM-PMAs [43].

The top and the bottom layers are typically based on metals such as gold (Au) [38,41], silver (Ag) [49], and aluminum (Al) [28], which are common materials for SPPs. Titanium nitride (TiN) [59,60], molybdenum (Mo) [43], and tungsten (W) [58], or highly-doped silicon (Si) [61], can be used for the bottom and top micropatches. Graphene can also be used for top micropatches in the IR wavelength region [62]. TiN or Mo have recently been used for thermal IR emitters due to requirements of high-temperature tolerance. However, absorbers require less temperature tolerance. Therefore, Al is widely employed due to its compatibility with the CMOS process and its low cost.

The middle layer is roughly classified into two groups: oxides or nitrides such as Al_2_O_3_ [41], SiO_2_ [40], CeO_2_ [63], SiN [28], and AlN [43], and semiconductors such as Si [64], germanium (Ge) [65], and ZnS [66]. Another material often used is MgF_2_ [33]. Phase transition materials of germanium antimony telluride [67] and VO_2_ [68] have been used for thermal switching. The loss is an important factor of the middle dielectric layer, as discussed in a later section. Lossless materials such as Si, Ge, ZnS, and CeO_2_ should be used for the IR wavelength region in the vicinity of 10 μm to maintain the linear tunability of the absorption wavelength. However, these materials are not compatible with the CMOS process. The alternative choice to avoid the influence of intrinsic loss materials is mushroom-PMAs, where small post structures connect the micropatches and the bottom layer without a continuous middle dielectric layer [69,70,71].

A perforated metal plate can be used as a top layer, which is a complementary structure of periodic micropatches [72] that provides optical properties similar to MIM-PMAs. However, there is less design flexibility due to the need for periodicity. 1D grating structures with ultra-narrow groove widths (ca. 100 nm) and high aspect ratios (>10) [16,18,73] can be classified as MIM-PMAs because such a slit is considered to be the waveguide, which is equivalent to the insulator layer in MIM structures [74,75]. However, high aspect ratios and narrow groove widths require complicated fabrication procedures. In this paper, these structures are excluded and instead focus is made on the conventional MIM-PMAs, shown as Figure 2. Other structures such as core-shell nanoparticles [76] and multi-flat-layer structures [77,78,79] can be considered to have a principle similar to that of MIM-PMAs.

## 3. Basic Optical Properties

### 3.1. Principle

**Figure 3:**
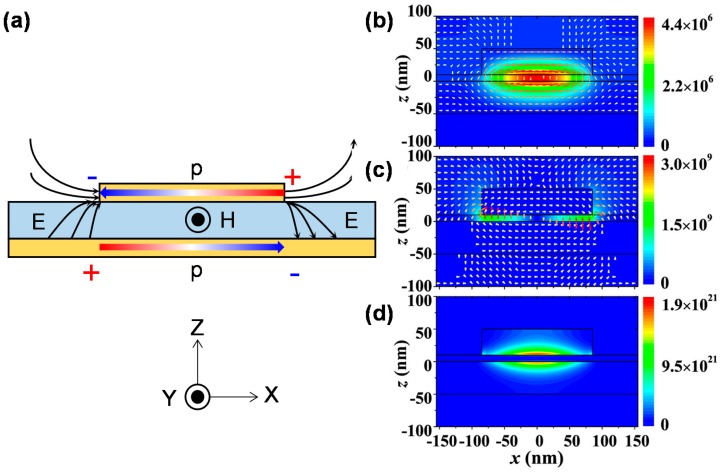
(**a**) Schematic illustration of the operating principle of MIM-PMAs. E, H, and p represent the electric displacement vector, magnetic field, and current, respectively. Calculated results: (**b**) magnetic field; (**c**) electric field; and (**d**) power distribution. The color maps represents the amplitude of each distribution. (**b**–**d**) are reprinted from Reference [41] with the permission of AIP Publishing.

Figure 3a shows the operating principle of MIM-PMAs for incident EM waves. Figure 3b–d shows the calculated electric and magnetic fields, and the power distribution of MIM-PMAs at the absorption wavelength, respectively [41].

As shown in Figure 3a, a pair of anti-parallel oscillating currents is induced in both the bottom layer and the top micropatches, and significant magnetic resonance is produced. Dipole electric resonance is formed accordingly between the edge of the micropatches and the near bottom layer. LSPPs are induced by the incident light at the absorption wavelength. This principle is confirmed by the calculated electromagnetic field distribution, as shown in Figure 3b–d [41]. The electric displacement vectors in the bottom layer and the micropatches are opposite to each other, which generates a strong magnetic response [80] (Figure 3b). Strong electric dipole resonances are observed at the sides of the micropatches (Figure 3c). The reflectance can be completely cancelled in the far field by the interference of these two dipoles due to the π shift phase [65]. Strong absorption is thus attributed to these localized magnetic and electric dipole resonances, which provide sufficient time to consume light energy by the ohmic losses in the metals (Figure 3d).

### 3.2. Wavelength Selectivity 

**Figure 4:**
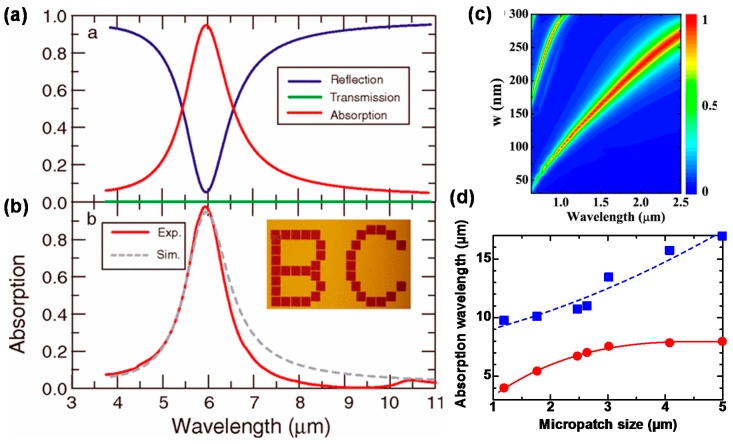
Calculated spectra for (**a**) absorption, reflection, and transmission; (**b**) Comparison of the measured and calculated absorption spectra; (**c**) Absorbance as a function of the wavelength and the micropatch size (w) in the near-IR wavelength region; (**d**) Relation between the micropatch size and the absorption wavelength in the IR wavelength region. (**a**,**b**) are adapted with permission from Reference [38]. © 2010 American Physical Society. (**c**) is reprinted from Reference [41] with the permission of AIP Publishing.

Figure 4a,b shows the calculated and measured optical properties of MIM-PMAs in IR wavelengths [38]. Wavelength selective absorption is clearly obtained at 6 μm, which is a typical wavelength-selective absorption property of MIM-PMAs. The main absorption wavelength is always longer than the period of the micropatches because wavelengths smaller than the period are diffracted. Figure 4c [41] and Figure 4d [81] show the calculated absorbance as a function of the wavelength and the micropatch size (w) in the near-IR wavelength region, as well as the measured relation between the micropatch size and the absorption wavelength in the IR wavelength region.

The absorption wavelength is almost proportional to the micropatch size in the near-IR wavelength region. In contrast, the relation between the micropatch size and the absorption wavelength is non-linear in the IR wavelength region, which is attributed to the loss of the middle dielectric layer [40]. Most oxides become lossy in the vicinity of 10 μm, where no absorption occurs, and this causes the non-linearity between the micropatch size and the absorption wavelength, as shown in Figure 4d. This is an important point for the design of MIM-PMAs for use at IR wavelengths. The thickness of the middle dielectric layer has less impact on the absorbance and the absorption wavelength, and can thus be optimized for the operating wavelength [41].

### 3.3. Incidence Angle Dependence

**Figure 5:**
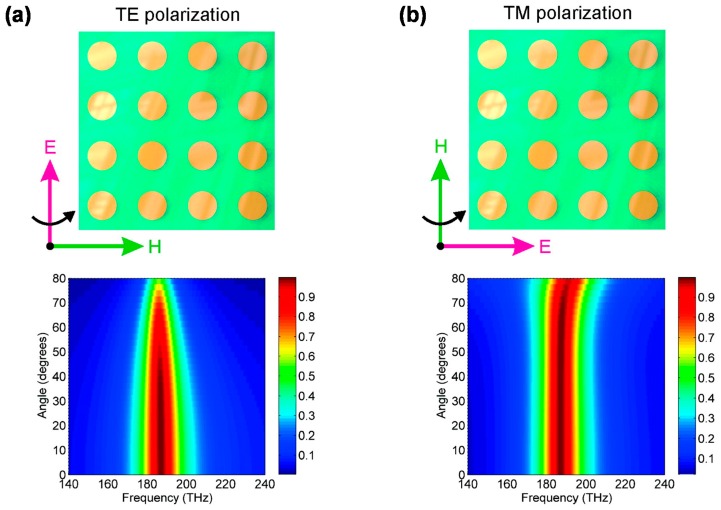
Calculated absorption for the (**a**) transverse-electric (TE) and (**b**) transverse-magnetic (TM) modes. Figures are adapted with permission from Reference [22]. © 2010 American Chemical Society.

Figure 5a,b shows the calculated incident angle dependence of the absorbance as a function of the wavelength for the transverse-electric (TE) and transverse-magnetic (TM) modes, respectively [22]. The calculated model is for an MIM-PMA with 2D circle-shaped micropatches.

Figure 5 shows that the absorption can be realized at almost the same wavelength for a wide range of incidence angle up to approximately 70° for both TE and TM modes. This property is attributed to the strong LSPPs, as shown in Figure 3. The incident angle independence is an important advantage for device applications such as solar cells, IR image sensors, and biological sensors.

### 3.4. Polarization Dependence

In this section, the coordinate system is set as shown in Figure 3c,d. When the electric field of the incident light is in the *x* or *y* direction, the absorption wavelength is defined by the side-length of the micropatches in the *x* or *y* direction, respectively [39]. Each side-length of the square-shaped micropatches in the *x* and *y* direction is the same. Thus, the absorption wavelength is also the same for each polarization. MIM-PMAs with this configuration are polarization insensitive. 

Different side lengths, such as ellipse [47] or asymmetric-cross-shaped [52] micropatches, produce dual band absorption, as discussed in the next section. The 1D periodic configuration shown in Figure 3b also produces two absorption modes. However, when one side-length is much longer than the other, the other absorbance is outside the operation wavelength region, which results in polarization-selective absorbers. Polarization-selective absorbers can be applied to IR polarimetric imaging [82,83] to enhance object recognition ability such as distinct human trace in a natural environment and human facial recognition [84].

### 3.5. Inductor-capacitor (LC) Circuit Model

**Figure 6:**
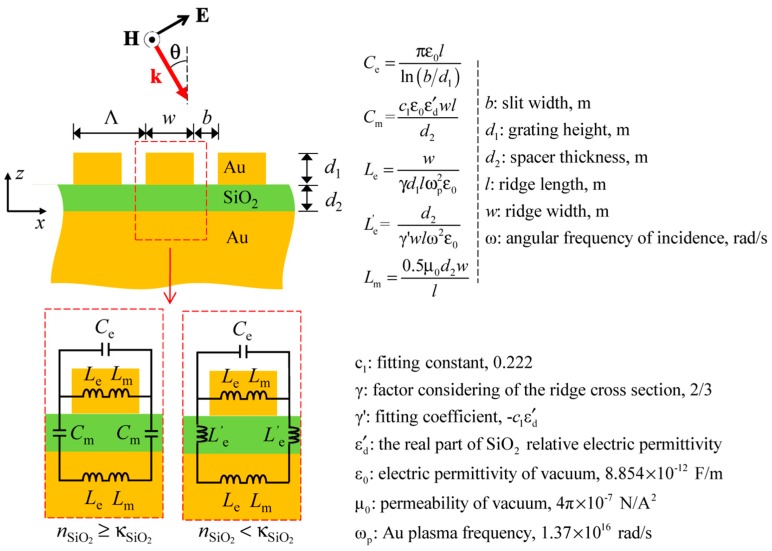
Schematic illustration of the LC equivalent circuit for MIM-PMAs. Figures were adapted with permission from Reference [40]. © 2013 Optical Society of America.

The operation principle of MIM-PMAs is sometimes explained using the LC equivalent circuit model. This may be due to the influence of CA absorbers mentioned in the introduction section. Figure 6 shows a schematic illustration of the LC equivalent circuit for MIM-PMAs [40,63]. Two models are considered, with or without the loss of the insulator layer. The frequency that gives a total impedance of zero is the absorption frequency.

## 4. Multi-Band and Broadband Operation 

**Figure 7:**
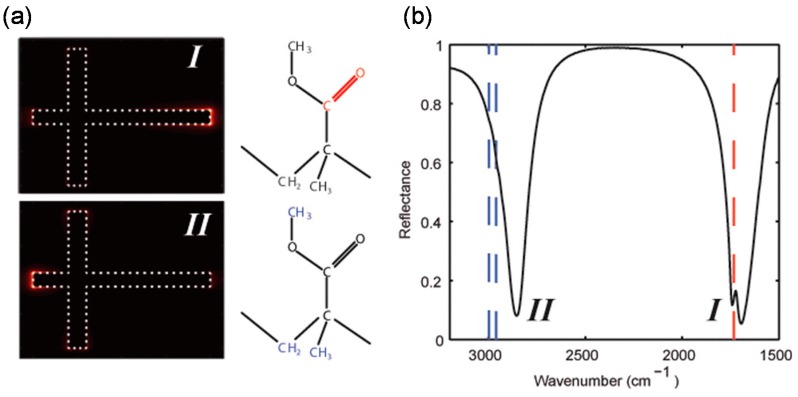

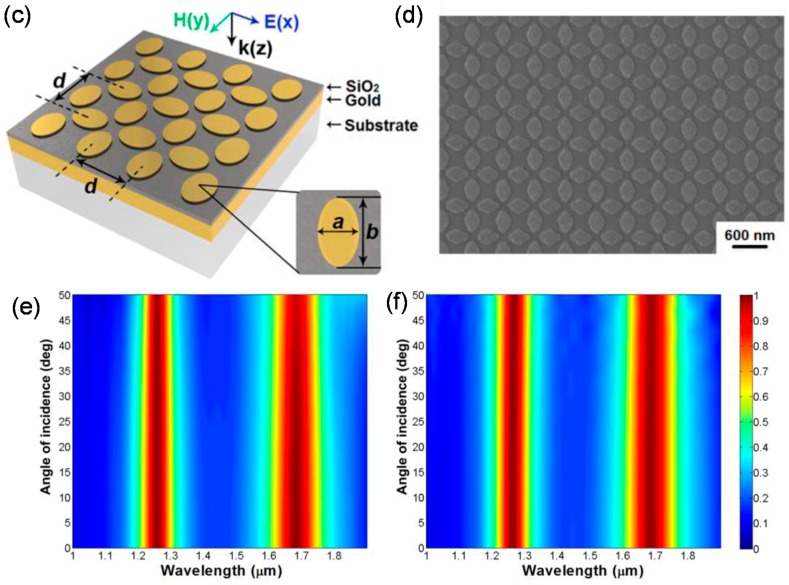
Schematic illustration of an MIM-PMA with asymmetric cross-shaped micropatches for dual-band operation. (**a**) Electric field distribution for modes I and II; and (**b**) the corresponding reflectance spectrum. (**c**) Schematic illustration and (**d**) SEM image of an MIM-PMA with an elliptical nanodisk array. Calculated absorbance for (**e**) TE and (**f**) TM modes. (**a**,**b**) are adapted with permission from Reference [52]. © 2012 American Chemical Society. (**c**–**f**) are adapted with permission from Reference [47]. © 2011 Optical Society of America.

The strategies of multi-band and broadband absorption are classified into three groups: asymmetrically-shaped [47,52,85,86,87,88,89] or multi-size [42,65,66,90,91,92,93,94] micropatches, multi-layers of MIM structure [95,96,97,98,99,100], and embedded in dielectric materials [57,101,102]. The first two are based on multi-resonance that produces multi-mode absorption. Each absorption mode becomes close, which results in broadband absorption [103]. Figure 7a–d shows MIM-PMAs with two-types of asymmetrically shaped micropatches, such as cross [52] and elliptical shapes [47], for dual-band operation. This dual-band absorption is designed in consideration of the polarization dependence for the two orthogonal directions.

**Figure 8:**
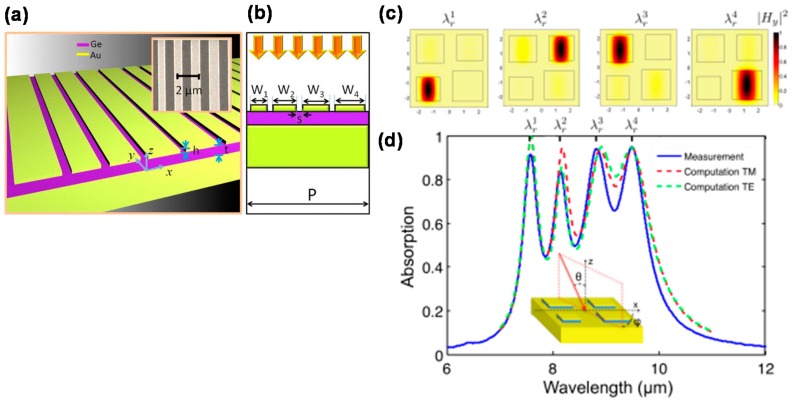
Schematic illustrations of MIM-PMAs with multi-size micropatches having (**a**,**b**) 1D and (**c**) 2D periodic configurations. (**d**) Calculated and measured absorption spectra for the 2D periodic configuration. (**a**,**b**) are reprinted from Reference [65] with the permission of AIP Publishing. (**c**,**d**) are adapted with permission from Reference [66]. © 2012 Optical Society of America.

Figure 8a,b shows oblique and the cross-sectional schematic illustrations of an MIM-PMA with 1D stripe-shaped multi-size-micropatches (w_1_ to w_4_), respectively [65]. Figure 8c,d shows a schematic illustration and magnetic field distribution of an MIM-PMA with 2D multi-size micropatches and the corresponding absorption spectrum, respectively [66]. The absorption spectrum is the summation of the absorption wavelengths generated by each micropatch resonator. Figure 8d shows that the distance between each resonant wavelength becomes close, which results in broadband absorption.

**Figure 9:**
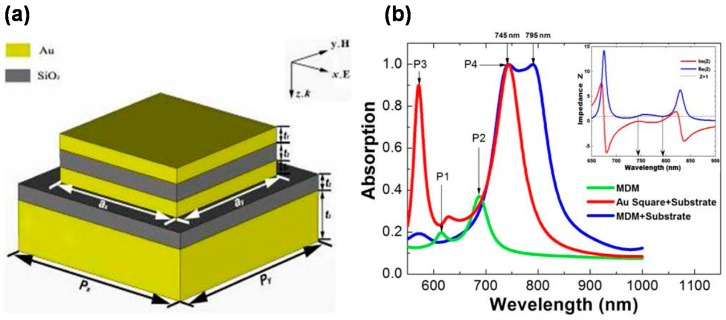
(**a**) Schematic illustration of a multi-layer MIM-PMA and (**b**) the calculated absorption spectrum. Figures are adapted with permission from Reference [95]. © 2012 Optical Society of America.

Figure 9a,b shows a schematic illustration of a multi-layer MIM-PMA and the calculated absorption spectrum, respectively [95]. Each MIM layer produces multi-plasmonic-resonances at different wavelengths and each resonance is coupled, so that broadband absorption occurs [103].

Broadband absorption is also achieved by MIM-PMAs with single or multi-layers embedded in lossy dielectrics such as amorphous Si [57] and SiN [101]. The resonances of MIM-PMAs can be broadened by lossy materials, which results in broad absorption. However, these structures increase the thickness and volume of the absorbers, and cause difficulties for fabrication. Care should be taken in applying them to practical devices by comparison with other convenient structures such as simple multi-flat-layer structures [77,78,79] or gold black [104,105] in terms of their thickness, ease of fabrication, and cost.

## 5. Advanced Structures and Applications

In this section, we briefly outline the advanced MIM-PMAs structures and applications other than absorbers to clarify the future research of MIM-PMAs. There has been growing interest in mainly three categories of advanced MIM-PMAs: flexible devices, the combination of graphene and other 2D materials, and metalenses.

**Figure 10:**
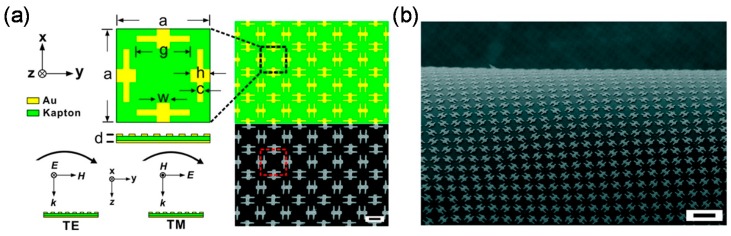
(**a**) Schematic illustration and (**b**) SEM image of a flexible MIM-PMA. Figures are adapted with permission from Reference [86]. © 2011 American Chemical Society.

One of the advanced structures is the flexible MIM-PMA [86,106,107], as shown in Figure 10 [86]. Flexible substrates such as Kapton film [86], polyethylene terephthalate (PET) [106] or polydimethylsiloxane (PDMS) [107] have enabled flexible MIM structures. MIM-PMAs coated on such flexible substrates can thus realize flexible and stretchable devices such as flexible solar cells, health care systems for the human body, and the cloaking of non-flat objects [108]. 

**Figure 11:**
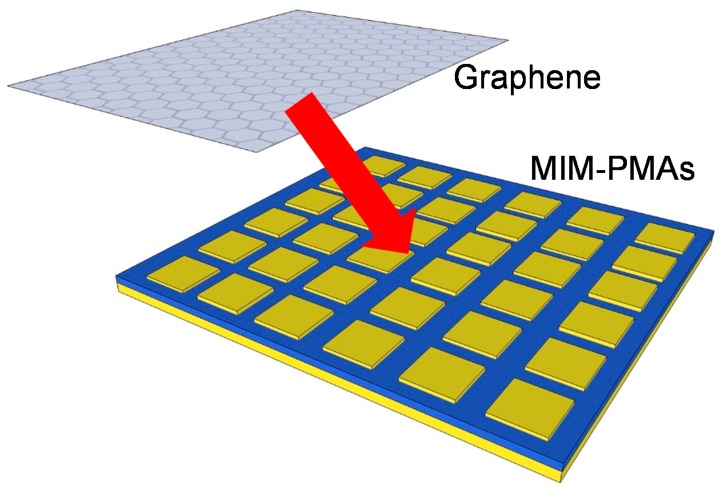
Schematic illustration of a graphene-coated MIM-PMA.

The combination of graphene [109] and other 2D atomic layer materials [110] such as MoS_2_ and WSe_2_ with MIM-PMAs [55,81,111,112,113,114,115] is also drawing significant interest because these 2D atomic layer materials can strongly interact with plasmonic resonance [116]. Figure 11 shows a schematic illustration of graphene coated on an MIM-PMA (GMIM-PMA). 

MIM-PMAs serve as a platform to enhance graphene absorption and realize high-performance graphene-based photodetectors [81,111,112,114]. The Fermi level of graphene can be electrically tuned according to the applied voltage; therefore, the absorption wavelength [117], reflection angle [118], and phase [119,120] can be electrically tuned by the applied voltage for graphene.

**Figure 12:**
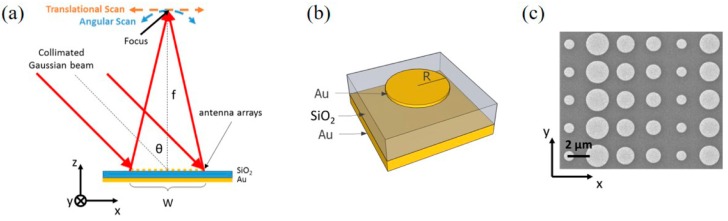
(**a**) Schematic illustration of a flat metalens using MIM-PMAs. (**b**) Schematic illustration of the unit cell of a reflector array lens. (**c**) SEM image of the metalens surface. Figures are adapted with permission from Reference [123]. © 2016 Optical Society of America.

Metalenses are a new type of flat lens based on geometrical phase control [121,122,123]. MIM-PMAs are considered as an array of optical resonators that can introduce a desired spatial profile of the optical phase and consequently mold the wavefront [123]. Figure 12 shows a schematic illustration of a metalens or reflector using MIM-PMAs [123]. The MIM-PMA structures control the reflection and their phase by phase gradient surface structures with different sized micropatches on the planar surface. Strong plasmonic resonance can change the phase of the reflection and thereby realize geometrical control of the phase. As a result, a flat metalens can be realized.

There are other rapidly growing research fields of MIM-PMAs, such as the non-linear response of second [124,125] and third [126] harmonic generation, and reflection control [127]. As discussed in this section, although MIM-PMAs are simple structures, they have significant potential for novel physics and applications.

## 6. Conclusions

MIM-PMAs have been reviewed here in terms of their structures, basic principles of absorption, materials used, absorption properties of incident angle and polarization dependence, and strategies of multiband or broadband operation to clarify the design strategies for visible and IR wavelengths. The same principles can be applied for a wide range of wavelength regions such as the ultraviolet [128], terahertz [129,130,131,132], and microwave [133,134,135] regions.

A single MIM structure can be considered as a single optical antenna with strong LSPPs. Therefore, MIM structures are free from the restriction of periodicity and beyond the diffraction limit, making them able to realize strong absorption and geometrical tunability of the absorption wavelength with a much thinner and smaller absorber volume than conventional EM absorbers. Such controllability opens up a new stage of EM absorber research and many novel applications are expected. 

In the future research of MIM-PMAs, flexible structures are also important to expand applications, such as health care devices for human sensing. The combination of new materials such as graphene and other 2D atomic materials with MIM-PMAs gives rise to the electrical tunability of the absorption wavelength, the phase, and the reflection angle because their optical constant can be tuned according to the applied voltage. MIM-PMAs can control other wavelengths than the absorption wavelength used. Thus, MIM-PMAs can be used for flat metalenses that can go beyond the diffraction limit.

We hope that this review paper will contribute to the development of advanced MM-PMAs and the expansion of their fields of application.

## Figures and Tables

**Table 1:** Properties of metals used in MIM-PMAs. Adapted with permission from Reference [43]. © 2017 American Chemical Society. (CMOS: complementary metal oxide semiconductor).

Material	Melting Point (°C)	Electrical Conductivity (×10^7^ S/m @20 °C)	Plasma Frequency (×10^15^ Hz)	Thermal Expansion Coefficient (CTE; ×10^−6^ K^−1^)	Young’s Modulus (E; GPa)	CMOS Compatibility
Al	660	3.5	3.57	24	70	yes
Au	1000	4.52	2.2	14	78	no
Pt	1770	0.944	1.25	8.8	168	no
TiN	2930	0.87	1.84	9.35	251	yes
Mo	2620	1.9	1.8	4.8	329	yes

**Table 2:** Properties of insulators used in MIM-PMAs. Adapted with permission from Reference [43]. © 2017 American Chemical Society.

Material	Melting Point (°C)	Young’s Modulus (E; GPa)	Poisson Ratio (μ)	CTE (×10^−6^ K^−1^)	Thermal Conductivity (W/m·K)
AlN	2200	344.8	0.287	4.6	285
SiO_2_	1600	70	0.17	0.5	1.4
Al_2_O_3_	2072	353.1	0.22	4.5	25.08
